# Evaluating the application of front-of-package labelling regulations to menu labelling in the Canadian restaurant sector using menu food label information and price (Menu-FLIP) 2020 data

**DOI:** 10.1017/S1368980024002143

**Published:** 2024-10-31

**Authors:** Hayun Jeong, Yahan Yang, Christine Mulligan, Mary R L’Abbé

**Affiliations:** Department of Nutritional Sciences, Temerty Faculty of Medicine, University of Toronto, Toronto, ON M5S 1A8, Canada

**Keywords:** Menu labelling, Restaurants, Front-of-package labelling

## Abstract

**Objective:**

To evaluate the application of front-of-package (FOP) labelling regulations to menu labelling in the Canadian restaurant sector by assessing the proportion of menu items that would be required to display the ‘high-in’ FOP symbol if the policy were extended to the restaurant sector.

**Design:**

Nutrition information of 18 760 menu items was collected from 141 chain restaurants in Canada. Menu items were evaluated using the mandatory FOP labelling regulations promulgated in Canada Gazette II by Health Canada in July of 2022.

**Setting:**

Chain restaurants with ≥20 establishments in Canada.

**Participants:**

Canadian chain restaurant menu items including beverages, desserts, entrées, sides and starters.

**Results:**

Overall, 77 % of menu items in the Canadian restaurant sector would display a ‘high-in’ FOP symbol. Among these menu items, 43 % would display ‘high-in’ one nutrient, 54 % would display ‘high-in’ two and 3 % would display ‘high-in’ all three nutrients-of-concern. By nutrient, 52 % were ‘high-in’ sodium, and 24 and 47 % were ‘high-in’ total sugars and saturated fat, respectively.

**Conclusions:**

Given the poor nutritional quality of restaurant foods, the current regulations, if applied to restaurant foods, would result in most menu items displaying a FOP symbol. Therefore, expanding the Canadian FOP labelling regulations to the restaurant sector can be key to ensuring a healthy food environment for Canadians. Furthermore, menu labelling along with other multi-faceted approaches such as reformulation targets are necessary to improve the dietary intake of Canadians from restaurant foods.

Non-communicable diseases (NCD), including diabetes and CVD, account for 74 % of deaths worldwide and 88 % of all deaths in Canada^(1)^. Diet has been established as a top modifiable risk factor for NCD^(2)^, with numerous studies showing the benefits of healthy dietary patterns rich in nutrient-dense foods^(3)^ as well as the detriments of unhealthy diets high in sodium, saturated fats and total sugars (i.e. nutrients-of-concern)^(4)^. Restaurant foods are not only anecdotally accepted as ‘unhealthy’ but have been consistently associated with excessive consumption of the nutrients-of-concern^(5)^, thereby contributing to an increased risk for obesity and NCD^(6,7)^. The most recent national nutrition survey data (CCHS 2015) indicate that more than half (54 %) of Canadians eat out once a week or more^(8)^, and as of 2019, the average Canadian household spends over one-quarter (26·9 %) of its food budget on restaurant foods^(9)^. With the COVID-19 pandemic coming to an end and restrictions lifted across the country, Statistics Canada also reported that restaurant sales have surpassed pre-pandemic levels, suggesting a new boom in dining out, as well as the growing trend of delivery and take-out services^(10)^. However, multiple studies from the past 10 years have shown that menu items in Canadian restaurants contain high levels of nutrients-of-concern^(11–13)^. While there are scarce data on the longitudinal changes in the nutritional quality of menu items, one study examined calorie levels among matched foods in chain restaurants from 2010 to 2017 and found no improvements but rather that they remained high^(13)^.

To counteract the potential harms associated with increased restaurant food consumption, countries have introduced menu labelling schemes to help consumers make informed and healthier choices in restaurants^(14)^. Currently, most jurisdictions have focused on calorie labelling, such as those mandated by the USA, the UK, New Zealand and Australia^(14,15)^. Studies from these countries investigating the policies’ outcomes repeatedly report an effective increase in public awareness of the energy content of menu items and healthier choices at restaurants^(16,17)^. In Canada, mandatory calorie labelling is only in force in Ontario under the Healthy Menu Choices Act 2015^(18)^. An assessment of the early impact of this policy on chain restaurants in Ontario found no significant nutritional changes in menu items after 1 year^(13)^. Nutrition interventions in the restaurant sector are one of the most important missing links in Canada’s fight to reverse obesity and diet-related NCD, as food policies in Canada, such as the Healthy Eating Strategy, have largely overlooked this important constituent of the Canadian diet^(19)^.

Evidence suggests menu labelling with calories alone is limited in its effects on consumers’ actual calorie intake or menu selection^(20,21)^. Therefore, countries have begun to translate front-of-package (FOP) labelling into interpretative menu labelling that leverages simple symbols representing the healthfulness of a menu item, which has been found to be effective in influencing consumer choice and their consumption of fewer calories^(21,22)^. This is congruent with the prepackaged food environment where there is an abundance of evidence to suggest that unless some form of interpretative nutrition information (i.e. via FOP labelling) is provided, not all consumers access the back of package nutrition information and do not improve their food choices^(23,24)^. Interpretative menu labelling that mirrors FOP labelling on prepackaged foods, however, is not mandated by any federal government yet. The USA endorses mandatory extensive provision of nutrition information for menu items, requiring chain restaurants to make their complete nutrition information available to the public on menu boards and websites^(25)^. Beyond providing nutrition information and calorie labelling, chain restaurants in New York and Philadelphia, USA, are also required to display a ‘saltshaker’ icon beside menu items containing more sodium than the federal daily recommended amount (%DV)^(26,27)^. In France, some chain restaurants are committed to displaying a Nutri-Score, an evidence-based FOP labelling system using colour and alphabet-graded coding^(28)^, on their menus to help consumers make informed and healthier choices at point-of-purchase^(29,30)^. Studies have demonstrated that FOP labelling on menu items in restaurants can have significantly positive effects on consumers’ food choices, although mixed effects were observed and more real-world studies are needed^(21)^. While Canada has recently promulgated a mandatory FOP labelling policy in Canada that mandates foods meeting or exceeding recommended thresholds for nutrients-of-concern for Canadians (i.e. sodium, saturated fats and total sugars) to display a ‘high-in’ FOP symbol by January of 2026^(31)^, the policy is limited to prepackaged foods and does not extend to restaurant foods.

As such, recent studies have only evaluated the generic and brand-name Canadian food composition databases composed of prepackaged foods and beverages against the new FOP labelling thresholds^(32,33)^. Given that the regulations assess foods against specific thresholds for nutrients known to be of concern in restaurant foods (sodium, saturated fats and sugars), an examination of the FOP labelling policy’s potential impact on restaurant foods in Canada is warranted. Therefore, the objectives of this study were (1) to assess the energy content and levels of nutrients-of-concern in Canadian chain restaurant menu items by major and submenu categories and (2) to assess the application of Canada’s FOP labelling regulations to menu labelling in the Canadian restaurant sector.

## Methods

### Study design

The nutrient content of chain restaurant menu items in Canada was analysed with a focus on energy and the nutrients-of-concern addressed by the FOP labelling policy (i.e. sodium, total sugars and saturated fats). Menu items were evaluated using the mandatory FOP labelling regulations promulgated in *Canada Gazette II* by Health Canada in July of 2022^(31)^.

### Menu-FLIP 2020

As part of the University of Toronto’s Food Label Information and Price (FLIP) database, Menu-FLIP contains comprehensive nutritional information for chain restaurants with 20 or more outlets across Canada and is updated every 3–4 years. Details for both the FLIP and Menu-FLIP databases have been published elsewhere^(12,34)^. In summary, Menu-FLIP 2020, collected in 2020, includes nutritional information for *n* 18 760 items from 141 top chain restaurants in Canada with publicly available data. Menu items were categorised into one of five major menu categories: (1) beverages, (2) desserts, (3) entrées, (4) sides and (5) starters. Items were then further categorised into subcategories, which are presented in the online supplementary material, Supplemental Table 1.

### Canadian mandatory FOP labelling regulations

The mandatory FOP labelling regulations promulgated in *Canada Gazette II* were used to identify menu items in the Menu-FLIP 2020 database that would carry a FOP symbol if the same regulations for prepackaged foods were applied to restaurant foods. The FOP labelling regulations mandate that all food and beverage products meeting or exceeding thresholds for sodium, sugars and/or saturated fat (i.e. nutrients-of-concern) to display a ‘high-in’ FOP symbol. Products are assessed on a per-nutrient basis, and the FOP symbol would display however many nutrients the product is ‘high-in’.

**Figure 1.**
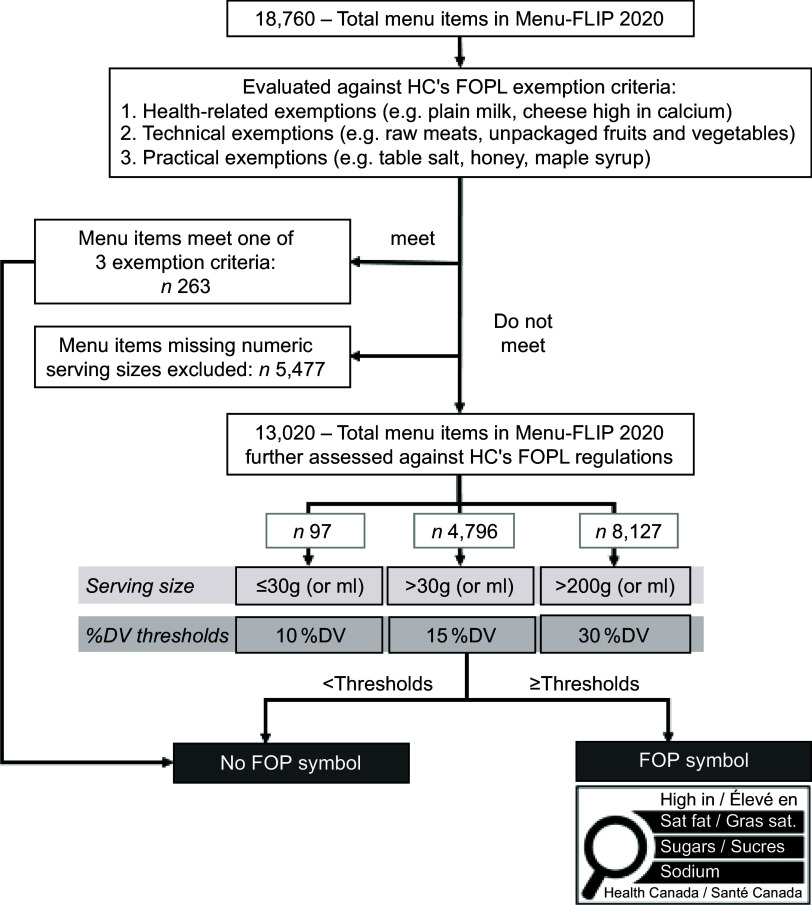
Flow chart of applying Canada’s front-of-package (FOP) labelling regulations to the total sample data for Menu-FLIP 2020. Menu items were first evaluated against exemption criteria. Items that did not meet the exemption criteria were further assessed against the FOP labelling thresholds for nutrients-of-concern (sodium, total sugars and saturated fat). For sodium, the thresholds were 230 mg, 345 mg and 690 mg for items with serving sizes of ≤30 g and >200 g, respectively. For total sugars, thresholds were 10 g, 15 g and 20 g; for saturated fat, thresholds were 2 g, 3 g and 6 g. Menu items not exceeding thresholds for all three nutrients would not display a ‘high-in’ FOP symbol. Items meeting or exceeding any of the thresholds would display the ‘high-in’ FOP symbol for 1–3 nutrients.

The thresholds are set based on the percent daily value (%DV) per stated serving size or reference amounts, whichever is greater, for each nutrient, reference amount and two different age groups (adults and children >4 years of age or children 1–4 years of age). Table 1 shows the summary of the thresholds for foods requiring a ‘high-in’ FOP symbol as outlined in *Canada Gazette II*. Most prepackaged foods (reference amount or serving size of >30 g) are assessed for the nutrients-of-concern at the 15 % DV threshold. Whereas foods with a smaller reference amount or serving size (≤30 g) and foods that are main dishes with a greater reference amount (≥200 g) are subject to 10 and 30 % DV thresholds, respectively. For main dish products intended solely for children 1–4 years of age, products with a reference amount of 170 g or more are subject to 30 % DV thresholds. Additionally, the regulations include three types of exemptions, whereby if a food product meets one of the three exemptions, it is exempt from the assessment and will not display a FOP symbol, regardless of its levels of nutrients-of-concern. The exemption criteria are presented in Fig. 1 and further described elsewhere^(35)^.

**Table 1.** Summary of Health Canada’s thresholds for foods requiring a ‘high-in’ FOP symbol as per the *Canada Gazette II* ^(31)^

Nutrient-of-concern	General prepackaged foods (>30 g or ml)	Prepackaged foods with small reference amount (≤30 g or ml)	Prepackaged main dishes^*^ with reference amount of ≥200 g	Prepackaged main dishes intended solely for children with reference amount of ≥170 g
Sodium	15 % DV = 345 mg	10 % DV = 230 mg	30 % DV = 690 mg	30 % DV = 690 mg
Sugars	15 % DV = 15 g	10 % DV = 10 g	30 % DV = 30 g	30 % DV = 30 g
Saturated Fat	15 % DV = 3 g	10 % DV = 2 g	30 % DV = 6 g	30 % DV = 6 g

FOP, front-of-package; DV, daily value.

* All menu items with serving sizes ≥200 g were assessed against the 30 % DV. As per Canada Gazette II, Health Canada has revised the ‘main dish’ definition to apply to products with a reference amount of 200 g or more (or 170 g or more in the case of products intended solely for children 1–4 years of age), given they contribute more nutrients to the individual diet than foods with reference amounts of < 200 g.

### Applying the front-of-package regulations to Menu-FLIP 2020

Menu items that would meet the exemption criteria were first determined, as these products would not be required to display a FOP symbol. The FOP thresholds are set based on Canada’s table of reference amounts for foods or stated serving sizes^(31)^. As there is no table of reference amounts set for restaurant foods, the serving sizes, when reported by each restaurant, were used to apply the regulations to menu items. To best adhere to the current regulations, menu items missing a numeric serving size (*n* 5477; 29·2 % of total items in Menu-FLIP 2020) were therefore excluded from the analysis. Following the exemptions and exclusions due to missing serving size values, the thresholds of 15, 10 and 30 % DV for most items, items with smaller serving sizes and items with greater serving sizes, respectively, were applied to menu items (Fig. 1).

### Statistical analysis

Analyses were performed using R Studio V2022·02·3. Descriptive statistics were performed, and mean and median levels (with 95 % CI) of energy and nutrients-of-concern by major and submenu categories were reported. The FOP thresholds for the nutrients-of-concern were applied to Menu-FLIP 2020 to assess the number and proportion of menu items that would be required to carry a ‘high-in’ FOP symbol. The number and type of ‘high-in’ nutrients (i.e. saturated fat, sodium and/or total sugars) that menu items would be required to display on the FOP symbol were also determined by major and submenu categories.

## Results

### Energy and nutrients-of-concern content of menu items by major and submenu category

The energy and nutrients-of-concern content by major and submenu categories are available in the online supplementary material, Supplemental Table 3. On average, starters had the highest calories per serving (mean = 665 kcal; CI = 625, 705) across major menu categories. Within starters, dips (mean = 1034 kcal; CI = 896, 1172) followed by fries and onion rings (mean = 900 kcal; CI = 806, 994), were the highest in calories. Calorie content was second highest in entrées (mean = 610 kcal; CI = 602, 619) and lowest in beverages (mean = 233 kcal; CI = 227, 239). Notably, poutine had the highest calories per serving across both submenu categories and overall (mean = 1835 kcal; CI = 1742, 1927). Sodium content was also the highest per serving in starters (mean = 1588 mg; CI = 1480, 1697) followed by entrées (mean = 1232 mg; CI = 1213, 1251) and sides (mean = 693 mg; CI = 663, 724). Across submenu categories, poutine (mean = 3373 mg, CI = 3195, 3552), miscellaneous (e.g. combos) in both entrées (mean = 2315 mg; CI = 2070, 2560) and starters (mean = 2218 mg; CI = 1645, 2793) had the highest levels of sodium per serving. On average, beverages were the highest in total sugars per serving (mean = 38·9 g; CI = 37·9, 40), followed by desserts (mean = 29·6 g; CI = 28·3, 30·9). Milkshakes/floats (mean = 75 g; CI = 70·9, 79·1) and juices/smoothies (mean = 47·8 g; CI = 45·8, 49·9) were the beverages with the highest total sugar levels, while frozen desserts (mean = 36·4 g; CI = 33·6, 39·2) had the highest total sugar levels among desserts. At the submenu category level, baked goods in entrées were also high in total sugars (mean = 44·9 g; CI = 41·5, 48·4). Starters had the highest saturated fat content per serving (mean = 10·1 g; CI = 9, 11·1), followed by entrées (mean = 9·6 g; CI = 9·4, 9·8). At the submenu category level, poutine (mean = 43 g; CI = 40·4, 45·5) and miscellaneous in entrées (mean = 23·5 g; CI = 18, 29) and dips in starters (mean = 21·9 g; CI = 17, 26·9) were highest in saturated fat per serving.

### Proportion of menu items that would carry the ‘high-in’ FOP symbol

**Figure 2.**
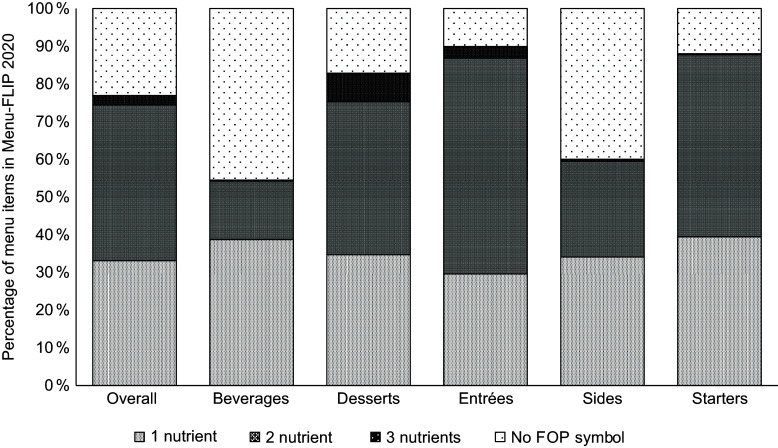
Percentage of menu items in Menu-FLIP 2020 that would display Health Canada’s ‘high-in’ front-of-package (FOP) symbol if the same regulations were applied to restaurant foods. Total *n* 13 283. Major menu categories were classified as either no FOP symbol or 1–3 nutrients (i.e. would display a FOP symbol for 1–3 nutrients-of-concern).

Figure 2 and online supplementary material, Supplemental Table 4 present the percentage and/or number of menu items in Menu-FLIP 2020 that would carry the ‘high-in’ FOP symbol by major and submenu categories, respectively. Of the 18 760 menu items, 1·4 % (*n* 263) would meet the exemption criteria of the FOP labelling regulations and would not be subject to displaying a FOP symbol. A total of 70·4 % (*n* 13 020) of the remaining items provided serving sizes and were further assessed against HC’s FOP labelling regulations (Fig. 1).

Overall, 77 % of menu items (*n* 10 217) would be required to display the ‘high-in’ FOP symbol, while 23 % (*n* 3066) would not be required to display the FOP symbol. Among menu items that would display a FOP symbol, 43 % (*n* 4400) would indicate one ‘high-in’ nutrient, 54 % (*n* 5491) would indicate two ‘high-in’ nutrients and 3 % (*n* 326) would indicate all three ‘high-in’ nutrients. The majority of items in all five major menu categories would carry a FOP symbol. The top two major menu categories with the highest proportion of items that would not display a FOP symbol were beverages (45 %, *n* 1537) and sides (40 %, *n* 575). The top major menu categories with the highest proportion of items that would display one ‘high-in’ nutrient were beverages (39 % of all beverages, *n* 1307) and starters (39 % of all starters, *n* 112). As there are many subcategories in beverages (i.e. alcohol, plain milk, water) that meet the exemption criteria (see online supplementary material, Supplemental Table 1), the beverages category had the highest proportion of items that would be exempt from displaying a FOP symbol. Among the remaining beverages, most were only ‘high-in’ total sugars, and thus, there was also a high proportion of items that would display a FOP symbol indicating one ‘high-in’ nutrient. The top category with the highest proportion of items that would display two ‘high-in’ nutrients was entrées (64 % of all entrées, *n* 3922) followed by starters (55 % of all starters; *n* 137). Desserts had the highest proportion of items that would indicate all three ‘high-in’ nutrients (9 % of all desserts, *n* 101).

**Figure 3.**
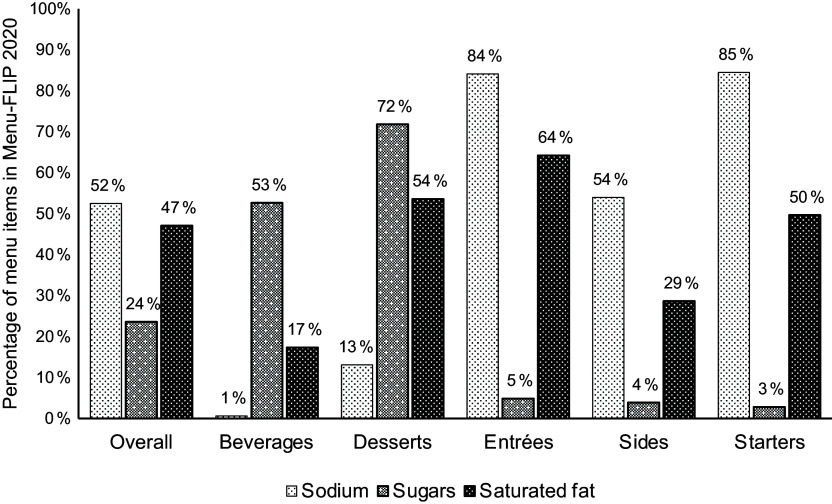
Percentage of menu items in Menu-FLIP 2020 that would display a ‘high-in’ front-of-package (FOP) symbol for each nutrient-of-concern: sodium, total sugars and saturated, according to Health Canada’s ‘high-in’ front-of-package labelling regulations for prepackaged foods, if the same regulations were applied to restaurant foods. Total *n* 13 283.

Figure 3 and online supplementary material, Supplemental Table 5 present the percentage and/or number of menu items in Menu-FLIP 2020 that would indicate each ‘high-in-‘ nutrient by major and submenu categories, respectively. Overall, 52 % (*n* 6972) of menu items in the analytical sample would indicate ‘high-in’ sodium, 24 % (*n* 3135) would indicate ‘high-in’ total sugars and 47 % (*n* 6253) would indicate ‘high-in’ saturated fat. Starters (85 %, *n* 240) and entrées (84 %, *n* 5760) had the highest proportion of items that would indicate ‘high-in’ sodium, while only 1 % (*n* 21) of beverages would indicate ‘high-in’ sodium. The top two major menu categories that would indicate ‘high-in’ total sugars were desserts (72 %, *n* 958) and beverages (53 %, *n* 1779), while 5 % or less of entrées (5 %, *n* 334), sides (4 %, *n* 56) and starters (3 %, *n* 8) would indicate ‘high-in’ total sugars. Entrées (64 %, *n* 4399) and desserts (54 %, *n* 715) were the top two major menu categories that would indicate ‘high-in’ saturated fat; 50 % of starters (*n* 141), 29 % of sides (*n* 412) and 17 % of beverages (*n* 586) would indicate ‘high-in’ saturated fat.

## Discussion

The primary objective of this study was to simulate the implementation of Canada’s FOP labelling regulations for prepackaged foods, in the restaurant sector by evaluating its application to restaurant menu items using the Menu-FLIP 2020 database. Overall, 77 % of menu items would carry the ‘high-in’ FOP symbol, if Canada’s FOP labelling regulations were applied to restaurant foods. Across major menu categories, most items in entrées, starters and sides would indicate ‘high-in’ sodium content. Most beverages and desserts would indicate ‘high-in’ total sugar content, and most desserts and entrées would indicate ‘high-in’ saturated fat content. Our findings demonstrate that a large proportion of restaurant foods sold in Canada are ‘high-in’ nutrients-of-concern. An extension of the FOP labelling regulations to restaurant foods may help inform consumers of the nutritional quality of their meals when eating out.

The results of the present study provide further evidence of the poor nutritional quality of restaurant foods, demonstrating the need for policies to help make healthier choices for consumers when eating out at restaurants. Sodium content was high for most entrées, starters and sides, with certain subcategories exceeding 100 % DV as recommended by Health Canada^(36)^, imposing a high risk for hypertension, heart disease and stroke^(4)^. Saturated fat content was also high for most entrées, desserts and starters, with the top contributing subcategory (poutine) exceeding 200 % DV. These results are alarming as international health organisations agree that saturated fat should be limited to <10 % of calories to lower the risk for CVD and the sodium DV is set at the Chronic Disease Risk Reduction Intakes (CDRR), rather than the recommended amount^(37)^. The WHO recommends a sugar intake of <10 % of energy intake to alleviate the health risks of excess sugar in predisposing consumers to dental cavities, overweight and obesity, which are risk factors for the development of diabetes, and further provides a conditional recommendation of <5 % of calories^(38)^. Our study shows that beverages and desserts available at restaurants in Canada, on average, have 39 g and 30 g of total sugars per serving, which amounts to 39 and 30 % DV, respectively. As such, an increased consumption of restaurant foods high in these three nutrients-of-concern is linked to poor diet quality, increased risk for overweight and obesity and consequential diet-related NCD^(4)^.

Consistent with previous studies indicating high levels of the nutrients-of-concern in restaurant foods, this study found that a large proportion of menu items in the Canadian restaurant sector (77 %) would display a ‘high-in’ FOP symbol if the same Canadian FOP labelling regulations were applied to restaurants. Dunford and colleagues conducted a similar study but in the Australian context by applying Australia’s Health Star Rating to restaurant foods, which provides an overall assessment of foods based on both nutrients-of-concern and nutrients that are encouraged in their diet^(39)^. Their results showed a mean score of 2·5 out of 5·0 stars, suggesting the technical feasibility of extending FOP labelling to fast foods. While this study echoes our results that demonstrate the potential for the Canadian ‘high-in’ FOP symbol to identify menu items that have excessive amounts of nutrients-of-concern, our findings raise the concern that if the majority of menu items would need to display a FOP symbol, consumers will be left with limited ‘healthy’ choices. Therefore, different labelling schemes may be necessary for the Canadian context to provide consumers with healthier options when eating out. Additionally, industry stakeholders identify the limited real estate of menus as a barrier to displaying menu labelling^(40)^. In combination with the high proportion of items that would require FOP symbols, this feasibility barrier is an important and pragmatic concern for the Canadian restaurant sector. However, there are real-world examples of FOP labelling implementation as menu labelling that counters industry arguments, demonstrating its feasibility and support from consumers. For example, France has successfully advanced from primarily implementing Nutri-Score on prepackaged foods to restaurants, such as McDonald’s due to its high consumer support^(29,30)^. Nutri-Score has been empirically demonstrated to significantly reduce consumers’ intake of calories, sugars and saturated fat, thereby improving the overall nutritional quality of meals^(41)^. Therefore, while there are potential challenges to applying the current FOP labelling regulations in the Canadian restaurant sector, the evidence from other countries suggests that with careful consideration of both consumer health benefits and industry concerns regarding feasibility, such regulations can be successfully implemented.

In addition, it is worth noting that the most prevalent type of ‘high-in’ nutrient in the Canadian restaurant sector was sodium. This is consistent with previous investigations reporting that the average Canadian restaurant menu items contained a day’s worth of the recommended intake of sodium and 22 % of menu items exceeded the daily limit of 2300 mg^(42)^, contributing to Canadians’ excess sodium intake. To tackle this public health concern, in 2012, Canada declared a sodium reduction goal to reduce the average sodium intake of Canadians from 3400 mg to 2300 mg/d by 2016^(43)^. As previous efforts only yielded modest results, Health Canada further updated its targets to reduce the amount of sodium in the prepackaged food supply as part of the Healthy Eating Strategy^(19)^. It is, however, concerning that these reduction targets and guidelines do not apply to restaurant foods that are significant contributors to Canadians’ sodium intake. Unless similar targets are established for restaurant foods, reducing individuals’ daily sodium intake will be challenging. An early study evaluating sodium changes in Canadian restaurants observed increases and decreases, concluding that voluntary industry efforts to decrease sodium levels in restaurant foods have produced inconsistent results^(42)^. The authors moreover highlighted that the reduction in some menu items demonstrated that sodium reduction is possible, while the increase in other items showcased the need for structured targets and timelines for effective sodium reduction in restaurant foods.

Research on the impact of information-based policies, such as FOP labelling and menu labelling, reveals a significant influence on consumer behaviour. These policies aim to provide clear and accessible nutritional information, thereby aiding consumers in making healthier dietary choices. Studies have shown that FOP labelling can lead to a noticeable shift in purchasing habits, guiding consumers towards healthier food products and/or steering them away from unhealthy options^(44)^. Their effectiveness has largely been attributed to their visibility that quickly captures the consumers’ attention and easy-to-interpret design that presents nutrition information and/or the healthfulness of a food or beverage in a way that can be easily understood at a glance^(24)^. Furthermore, with continual exposure to easy-to-interpret information, consumers’ nutritional knowledge and attitudes and trust in and use of the labels increase, which can result in sustained changes in their food purchasing behaviour and overall diet^(24)^. Therefore, if applied to menu labelling, there is potential for FOP labelling to equip consumers to make healthier choices when eating out as well^(45)^. According to a Cochrane review, evidence suggests that when calorie content is presented alone, it does not significantly affect consumers’ actual food choices and caloric intake, but when calorie labels are presented alongside interpretive information (i.e. FOP labelling), it can improve consumers’ actual food choices and consumption at restaurants^(21)^. However, a critical aspect of the Canadian FOP labelling’s impact lies in consumers’ comprehension of the absence of these labels. While consumers cannot discern at a glance whether foods or beverages do not display a FOP symbol because they are exempt from the regulations or if they are naturally lower in nutrients-of-concern, this distinction may not be great as most exempted foods are those recommended by Canada’s Food Guide (i.e. some dairy products) and foods already exempted from nutrition labelling (e.g. raw meats, fresh fruits and vegetables) or those with an obvious nutrient content (e.g. table salt). Therefore, ensuring clear communication and education during the implementation of FOP labelling regulations is important for maintaining the policy’s integrity and maximising its positive influence on consumer behaviour.

There is also some literature that suggests menu labelling can encourage reformulation of restaurant foods to be lower in calories and nutrients-of-concern. Prior to the mandatory calorie labelling policy implemented in the USA in 2016, researchers compared differences in calorie counts of food items between restaurants that voluntarily adopted national menu labelling and those that did not^(46)^. They found that the mean per-item calorie content was consistently lower for restaurants that voluntarily posted calorie information on their menus, suggesting calorie labelling may significantly impact restaurant menu items available in restaurants by encouraging reformulation and the introduction of lower-calorie items. The same research team conducted another longitudinal analysis of US restaurant data from 2012 to 2018 and observed that newly introduced menu items in large chain restaurants continued to decline in calories through 2018^(47)^. However, changes in nutrient content were sporadic and not clearly indicative of improved dietary quality, likely due to calorie labelling incentivising manufacturers to reduce calories without incentives to improve overall nutritional quality. Therefore, with the Canadian FOP labelling system indicating high levels of nutrients-of-concern, it may incentivise the reformulation of menu items to lower these nutrient levels. Moreover, it has been demonstrated that one pathway by which FOP labelling helps consumers make healthier choices is through stimulating healthier food production and manufacturer-driven product reformulation^(24)^. Consequently, policymakers should prioritise implementing menu labelling, potentially by applying a FOP labelling system, to help consumers make informed and healthier choices, incentivise menu reformulations and serve as a first step towards establishing mandatory sodium targets for restaurant foods.

This is the first study to date that examined the potential impact of the recently passed Canadian FOP labelling regulations on the Canadian restaurant sector if the same regulations were to be applied. With the lack of regulations around restaurant foods, our findings contribute to a body of evidence supporting the poor nutritional quality of restaurant foods, and furthermore, the need for policy action to help consumers make healthier choices when eating out at restaurants. Our study also used a nationally representative large database covering the majority of the top restaurants that represent more than 70 % of the 2020 market share in the chain restaurant sector in Canada including both fast food and sit-down restaurant chains^(48)^. However, there are several limitations to this study. First, as the most recent Menu-FLIP data were collected in 2020, it is possible that there have been some changes in the menu items available since then. Newly introduced or reformulated menu items would not have been captured, and items removed from the restaurants would have been included in this analysis. Furthermore, the excluded samples without serving sizes (29 %) could have created bias, as the excluded items may have been systematically different (i.e. healthier or unhealthier) from those that did provide information. A separate sensitivity analysis was conducted for menu items missing serving sizes, applying a 15 % DV threshold to desserts and sides that had mean serving sizes <200 g and a 30 % DV threshold to beverages, entrées and starters that had mean serving sizes ≥200 g (see online supplementary material, Supplemental Table 6). The heterogeneity across and within categories as well as the limitations of assigning thresholds based on serving size assumptions justified the exclusion of items with missing serving size in the analysis. As it has been shown that serving size, rather than calorie density is the major determinant of energy content^(49)^, the lack of regulation on standardising and reporting serving size could lead to serving size manipulation that reports nutrition values lower than regularly consumed (e.g. ½ of a muffin). Therefore, requiring serving sizes and nutrition information to be based on amounts that are customarily consumed, similar to Health Canada’s table of reference amounts for prepackaged foods, would allow for better future surveillance and research on the nutritional quality of restaurant foods. Furthermore, this analysis focused on large chain restaurants, and therefore, the generalisability to the full restaurant sector including independent restaurants can be limited, although the database captured over 70 % of the market share of the restaurant sector in Canada. The accuracy of the results also depends on the accuracy of the data provided by the restaurants, although they are required to provide up-to-date and accurate information, as required by the Food and Drugs Act, Section 5(1)^(50)^.

There is a lack of policy and labelling regulations in the restaurant sector in Canada and across the globe. Canada’s ‘high-in’ FOP symbol has much potential to be applied to restaurant foods, which are currently excluded from the regulations. Applying the current regulations would result in the majority of menu items displaying at least one FOP ‘high-in’ symbol. Thus, further development of global best practices for policies regulating and monitoring the restaurant sector is needed to help consumers identify healthier choices when eating at restaurants. The results moreover demonstrated an urgent need for improving the poor nutritional quality of restaurant foods through a multi-faceted approach such as menu labelling and structured targets for the nutrients-of-concern in restaurant foods. Further research into more restaurant-specific labelling and consumer research would help advance policy in this field.

## Supporting information

Jeong et al. supplementary material Jeong et al. supplementary material

## Supplementary material

For supplementary material accompanying this paper, visit https://doi.org/10.1017/S1368980024002143
