# Supplementary material for: Evaluating the application of front-of-package labelling regulations to menu labelling in the Canadian restaurant sector using menu food label information and price (Menu-FLIP) 2020 data
Source: Public Health Nutr. 2024 Oct 31;27(1):e238. doi: 10.1017/S1368980024002143 (PMC11645116; doi:10.1017/S1368980024002143)
Supplement: Jeong et al. supplementary material [file S1368980024002143sup001.docx]

**Table of Contents**

[**Supplementary Table 1.** Major and sub menu categories in Menu-FLIP 2020 2](#_Toc172644382)

[**Supplementary Table 2.** Serving size and energy content in menu Menu-FLIP 2020 by major and sub menu categories. 3](#_Toc172644383)

[**Supplementary Table 3.** Levels of nutrients-of-concern in menu items in Menu-FLIP 2020 by major and sub menu categories. 6](#_Toc172644384)

[**Supplementary Table 4.** The number and percentage of menu items in Menu-FLIP 2020 that would display the ‘high-in’ FOP symbol by major and sub menu categories, if the Canadian FOP labelling regulations were applied to restaurant foods. 9](#_Toc172644385)

[**Supplementary Table 5.** The number and percentage of menu items in Menu-FLIP 2020 that would display each ‘high-in’ nutrients-of-concern, if the Canadian FOPL regulations were applied to restaurant foods. 12](#_Toc172644386)

[**Supplementary Table 6.** Sensitivity analysis of the percentage of menu items in Menu-FLIP 2020 that would display a ‘high-in’ FOP symbol determined using an assessment that was based on major menu categories. 14](#_Toc172644387)

**Supplementary Table 1.** Major and sub menu categories in Menu-FLIP 2020.

| **Major Menu Category** | **Sub Menu Category** |  | **Major Menu Category** | **Sub Menu Category** |
| --- | --- | --- | --- | --- |
| Beverages  (n=3655) | Alcohol^*^ (n=106) |  |  | Soup (n=224) |
|  | Classic coffee/teas (n=365) |  |  | Tacos/burritos (n=476) |
|  | Flavoured coffee/teas (n=1053) |  |  | Vegetarian (n=281) |
|  | Flavoured milk (193) |  | Sides  (n=2175) | Bagels (n=89) |
|  | Juices/smoothies (n=831) |  |  | Breads (n=274) |
|  | Kids (n=57) |  |  | Breakfast (n=214) |
|  | Milkshakes/floats (n=57) |  |  | Cheese^*^ (n=61) |
|  | Plain milk^*^ (n=39) |  |  | Fries & Onion rings (n=347) |
|  | Soft drinks (n=751) |  |  | Fruit^*^ (n=22) |
|  | Water^*^ (n=42) |  |  | Kids (n=86) |
| Desserts  (n=1755) | Cookies (n=128) |  |  | Meat (n=83) |
|  | Donuts (n=137) |  |  | Misc. (n=38) |
|  | Frozen desserts (n=602) |  |  | Other baked goods (n=94) |
|  | Kids (n=105) |  |  | Pasta (n=23) |
|  | Misc. (n=14) |  |  | Potatoes (non-fried) (n=100) |
|  | Muffins (n=94) |  |  | Poultry (n=159) |
|  | Other baked goods (n=675) |  |  | Rice (n=48) |
| Entrées  (n=10713) | B-Baked goods^†^ (n=351) |  |  | Salad (n=246) |
|  | B-Egg plates^†^ (n=536) |  |  | Seafood (n=50) |
|  | B-Misc.^†^ (n=116) |  |  | Soup (n=98) |
|  | B-Sandwich/wrap/toast^2^ (n=313) |  |  | Vegetables (n=143) |
|  | Burgers (n=578) |  | Starters  (n=462) | Breads (n=43) |
|  | Hotdogs (n=47) |  |  | Cheese^*^ (n=20) |
|  | Kids (n=449) |  |  | Dips (n=42) |
|  | Meat only (n=156) |  |  | Fries & Onion rings (n=50) |
|  | Meat plate (n=294) |  |  | Meat (n=27) |
|  | Misc. (n=34) |  |  | Misc. (n=28) |
|  | Other baked goods (n=114) |  |  | Poultry (n=70) |
|  | Pasta (n=257) |  |  | Salad (n=21) |
|  | Pizza (n=2995) |  |  | Seafood (n=72) |
|  | Poultry only (n=325) |  |  | Soup (n=57) |
|  | Poultry plate (n=355) |  |  | Vegetables (n=32) |
|  | Poutine (n=314) |  |  |  |
|  | Salad (n=444) |  |  |  |
|  | Sandwiches/wraps (n=1324) |  |  |  |
|  | Seafood only (n=127) |  |  |  |
|  | Seafood plate (n=603) |  |  |  |

Abbreviations: FLIP, Food Label Information and Price database; ^*^Categories that meet the exemption criteria outlined in the Canada Gazette II; items in these categories were exempted from further analysis. ^†^Breakfast items.

## **Supplementary Table 2.** Serving size and energy content in menu Menu-FLIP 2020 by major and sub menu categories.

|  |  |  | **Serving size (g)** | | |  | **Calories (kcal) per serving** | | |
| --- | --- | --- | --- | --- | --- | --- | --- | --- | --- |
| **Menu Category** | ***Total n*** |  | **n** | **Mean (95% CI)** | **Median** |  | **n** | **Mean (95% CI)** | **Median** |
| **Overall** | 18760 |  | 13203 | 323.7 (319.8, 327.7) | 275 |  | 18760 | 483.1 (477.3, 489) | 3630 |
| **Beverages** | 3655 |  | 3320 | 474.8 (468.2, 481.3) | 467 |  | 3655 | 233 (227.3, 238.7) | 210 |
| Alcohol^*^ | 106 |  | 65 | 333.3 (296.6, 369.9) | 358.44 |  | 106 | 204.8 (178.6, 231) | 162 |
| Classiccoffee/Teas | 365 |  | 314 | 390.7 (368.5, 413) | 364.62 |  | 365 | 32.4 (26.1, 38.7) | 5 |
| Flavouredcoffee/Teas | 1053 |  | 984 | 456.2 (446.7, 465.7) | 464 |  | 1053 | 263 (254.2, 271.7) | 250 |
| Flavouredmilk | 193 |  | 176 | 383.2 (363.7, 402.6) | 399.04 |  | 193 | 346.9 (327.1, 366.8) | 340 |
| Juices/Smoothies | 831 |  | 740 | 506.9 (492.2, 521.7) | 478 |  | 831 | 246.8 (237.5, 256) | 220 |
| Kids | 57 |  | 39 | 251.4 (227.5, 275.2) | 238.96 |  | 57 | 159.2 (139.4, 179.1) | 150 |
| Milkshakes/Floats | 218 |  | 205 | 429.2 (409.8, 448.7) | 411 |  | 218 | 558.3 (529.1, 587.5) | 540 |
| Plainmilk^*^ | 39 |  | 28 | 354.5 (301.9, 407.2) | 341.96 |  | 39 | 159.3 (132.4, 186.1) | 140 |
| Softdrinks | 751 |  | 734 | 567.1 (551.8, 582.5) | 591 |  | 751 | 175.7 (166.6, 184.8) | 160 |
| Water^*^ | 42 |  | 35 | 467.8 (417.4, 518.2) | 515 |  | 42 | 5.6 (, 1.9, 13.1) | 0 |
| **Desserts** | 1755 |  | 1334 | 134 (128.7, 139.2) | 100 |  | 1755 | 351.3 (339.1, 363.5) | 290 |
| Cookies | 128 |  | 90 | 58.1 (47.3, 69) | 45 |  | 128 | 279.3 (244.9, 313.6) | 230 |
| Donuts | 137 |  | 75 | 65.5 (57.2, 73.8) | 64 |  | 137 | 270.8 (245.8, 295.7) | 280 |
| Frozendesserts | 602 |  | 520 | 165.4 (155.4, 175.3) | 116.0 |  | 602 | 307.8 (282.8, 332.8) | 148.3 |
| Kids | 105 |  | 89 | 102.6 (88.9, 116.3) | 74 |  | 105 | 222.2 (192.9, 251.5) | 170 |
| Misc. | 14 |  | 8 | 97.7 (20, 175.3) | 50 |  | 14 | 334.8 (208.1, 461.4) | 275 |
| Muffins | 94 |  | 63 | 125.3 (120.3, 130.3) | 124 |  | 94 | 413.4 (396.7, 430.2) | 420 |
| Otherbakedgoods | 675 |  | 489 | 132.4 (125, 139.9) | 105 |  | 675 | 431.9 (413.7, 450.1) | 380 |
| **Entrées** | 10713 |  | 6848 | 316.1 (310.5, 321.6) | 249 |  | 10713 | 610 (601.5, 618.5) | 495 |
| Breakfast Bakedgoods | 351 |  | 133 | 377.8 (339, 416.5) | 365 |  | 351 | 778.5 (745.2, 811.8) | 740 |
| Breakfast Eggplates | 536 |  | 237 | 571.4 (549.7, 593.1) | 565 |  | 536 | 935.6 (904.9, 966.2) | 922.5 |
| Breakfast Misc. | 116 |  | 72 | 520.1 (482.9, 557.3) | 533.5 |  | 116 | 824.1 (758.6, 889.7) | 754.5 |
| Sandwich/Wrap/Toast | 313 |  | 187 | 300.8 (274.3, 327.3) | 233 |  | 313 | 660.3 (625.3, 695.3) | 560 |
| Burgers | 578 |  | 347 | 331.8 (317.1, 346.4) | 326 |  | 578 | 841.7 (814.3, 869.1) | 820 |
| Hotdogs | 47 |  | 29 | 165.9 (144.1, 187.7) | 145 |  | 47 | 461.4 (408.1, 514.7) | 420 |
| Kids | 449 |  | 264 | 211.7 (199.6, 223.8) | 192 |  | 449 | 425.7 (404.7, 446.7) | 372 |
| Meatonly | 156 |  | 123 | 392.6 (357.4, 427.8) | 374 |  | 156 | 756 (692.4, 819.6) | 660 |
| Meatplate | 294 |  | 163 | 489.9 (456.1, 523.7) | 454 |  | 294 | 822.8 (777.4, 868.2) | 765.5 |
| Misc. | 34 |  | 15 | 549.7 (478.6, 620.8) | 618 |  | 34 | 995.2 (867.7, 1122.8) | 895 |
| Otherbakedgoods | 114 |  | 81 | 318.3 (276.7, 359.9) | 297 |  | 114 | 741.9 (663.5, 820.4) | 680.5 |
| Pasta | 257 |  | 219 | 536.2 (506.6, 565.7) | 542 |  | 257 | 800.2 (761.3, 839.1) | 780 |
| Pizza | 2995 |  | 2022 | 131 (127.5, 134.4) | 110 |  | 2995 | 320.2 (310.9, 329.6) | 250 |
| Poultryonly | 325 |  | 260 | 264.5 (241.6, 287.4) | 217 |  | 325 | 587 (543.6, 630.3) | 470 |
| Poultryplate | 355 |  | 195 | 501.6 (474.2, 529) | 470 |  | 355 | 838.4 (798.9, 877.9) | 771 |
| Poutine | 314 |  | 286 | 837.6 (797.2, 878) | 737.5 |  | 314 | 1834.5 (1741.8, 1927.2) | 1680 |
| Salad | 444 |  | 286 | 347.3 (333.7, 360.9) | 359 |  | 444 | 492.9 (470, 515.8) | 458 |
| Sandwiches/Wraps | 1324 |  | 868 | 345.1 (334.7, 355.5) | 305 |  | 1324 | 668.9 (652.7, 685.1) | 600 |
| Seafoodonly | 127 |  | 47 | 298.4 (247, 349.8) | 238 |  | 127 | 534 (475, 593) | 450 |
| Seafoodplate | 603 |  | 399 | 312 (296.3, 327.8) | 255 |  | 603 | 618.3 (588.3, 648.2) | 530 |
| Soup | 224 |  | 183 | 332 (313.3, 350.7) | 292.52 |  | 224 | 216.7 (195.9, 237.4) | 170 |
| Tacos/Burritos | 476 |  | 274 | 332.9 (311.5, 354.3) | 298 |  | 476 | 626.7 (598.3, 655) | 587 |
| Vegetarian | 281 |  | 158 | 438.8 (406.5, 471.1) | 462.5 |  | 281 | 566.9 (534, 599.8) | 540 |
| **Sides** | 2175 |  | 1417 | 190.9 (182.1, 199.7) | 149 |  | 2175 | 346.5 (333.2, 359.7) | 250 |
| Bagels | 89 |  | 62 | 128.8 (122, 135.7) | 123 |  | 89 | 342.7 (326.2, 359.1) | 330 |
| Breads | 274 |  | 181 | 68 (62, 73.9) | 54 |  | 274 | 229.9 (207.1, 252.8) | 170 |
| Breakfast | 214 |  | 96 | 127.8 (109.2, 146.4) | 100 |  | 214 | 245.1 (226.4, 263.7) | 220 |
| Cheese* | 61 |  | 48 | 85.2 (60.7, 109.7) | 45 |  | 61 | 273.3 (190.7, 356) | 120 |
| Fries&Onionrings | 347 |  | 252 | 342.3 (312.2, 372.4) | 278 |  | 347 | 697.9 (653.9, 741.9) | 605 |
| Fruit* | 22 |  | 11 | 186.7 (123.9, 249.5) | 174 |  | 22 | 117.2 (76.8, 157.6) | 95 |
| Kids | 86 |  | 58 | 110.1 (97.5, 122.6) | 100.025 |  | 86 | 194 (161.7, 226.3) | 145 |
| Meat | 83 |  | 44 | 83.5 (64.5, 102.5) | 64 |  | 83 | 226.1 (191.2, 261) | 190 |
| Misc. | 38 |  | 26 | 124 (78.9, 169.1) | 79.5 |  | 38 | 256.9 (202.5, 311.2) | 220 |
| Otherbakedgoods | 94 |  | 41 | 137.2 (83.9, 190.5) | 79 |  | 94 | 409 (340.8, 477.2) | 310 |
| Pasta | 23 |  | 13 | 252.8 (193.4, 312.1) | 235 |  | 23 | 543.2 (394.1, 692.3) | 480 |
| Potatoes (non-fried) | 100 |  | 70 | 288.1 (249.6, 326.7) | 270.5 |  | 100 | 380.8 (322, 439.5) | 330 |
| Poultry | 159 |  | 116 | 232 (202.1, 261.8) | 215.5 |  | 159 | 488.7 (425.8, 551.7) | 380 |
| Rice | 48 |  | 31 | 212.9 (171.4, 254.4) | 174 |  | 48 | 339.2 (282.2, 396.2) | 260 |
| Salad | 246 |  | 166 | 170.3 (156.4, 184.3) | 150 |  | 246 | 231 (208.9, 253.2) | 200 |
| Seafood | 50 |  | 42 | 125.5 (101.2, 149.7) | 104 |  | 50 | 212.7 (172.8, 252.7) | 200 |
| Soup | 98 |  | 68 | 273.5 (246.2, 300.8) | 230.892 |  | 98 | 194.7 (174.2, 215.1) | 170 |
| Vegetables | 143 |  | 92 | 190.4 (164.1, 216.6) | 148 |  | 143 | 219.8 (188.6, 250.9) | 167 |
| **Starters** | 462 |  | 284 | 296.1 (278.9, 313.3) | 288 |  | 462 | 664.9 (624.8, 704.9) | 591 |
| Breads | 43 |  | 31 | 239.5 (182.2, 296.8) | 230 |  | 43 | 659.4 (520.8, 798) | 550 |
| Cheese* | 20 |  | 11 | 228 (159.9, 296.1) | 231 |  | 20 | 619.8 (505.6, 733.9) | 590 |
| Dips | 42 |  | 26 | 396.5 (334.5, 458.5) | 374.5 |  | 42 | 1034.1 (896.4, 1171.8) | 930 |
| Fries&Onionrings | 50 |  | 27 | 347.3 (290, 404.5) | 304.0 |  | 50 | 899.7 (805.8, 993.6) | 840.9 |
| Meat | 27 |  | 16 | 345.8 (262.4, 429.1) | 363 |  | 27 | 782.9 (658.6, 907.2) | 788 |
| Misc. | 28 |  | 15 | 320.4 (220.3, 420.5) | 322 |  | 28 | 864.4 (678.1, 1050.8) | 842.5 |
| Poultry | 70 |  | 41 | 317.2 (262.6, 371.9) | 284 |  | 70 | 828.5 (709.2, 947.8) | 683 |
| Salad | 21 |  | 12 | 151.3 (138.5, 164) | 150.5 |  | 21 | 213 (139, 287) | 220 |
| Seafood | 72 |  | 40 | 266.6 (236.6, 296.5) | 282 |  | 72 | 558.8 (490.2, 627.4) | 550 |
| Soup | 57 |  | 51 | 288.8 (277.1, 300.4) | 293.48 |  | 57 | 208.6 (185.6, 231.6) | 178 |
| Vegetables | 32 |  | 14 | 280.4 (181.9, 378.8) | 293 |  | 32 | 564.9 (449.4, 680.3) | 508 |

Abbreviations: FLIP, Food Label Information and Price database; ^*^Categories that meet the exemption criteria outlined in the Canada Gazette II; items in these categories were exempted from further analysis.

## **Supplementary Table 3.** Levels of nutrients-of-concern in menu items in Menu-FLIP 2020 by major and sub menu categories.

Abbreviations: FLIP, Food Label Information and Price database; ^*^Categories that meet the exemption criteria outlined in the Canada Gazette II; items in these categories were exempted from further analysis.

**Supplementary Table 4.** The number and percentage of menu items in Menu-FLIP 2020 that would display the ‘high-in’ FOP symbol by major and sub menu categories, if the Canadian FOP labelling regulations were applied to restaurant foods. The proportions of menu items that would be high in 1-3 nutrients-of-concern are also presented.

|  |  |  |  | Number of 'high-in' nutrients | | |
| --- | --- | --- | --- | --- | --- | --- |
| Menu Category | Total n | No FOP symbol | FOP symbol | 1 | 2 | 3 |
| Overall | **13283** | **2803 (21.1%)** | **10217 (76.9%)** | **4400 (33.1%)** | **5491 (41.3%)** | **326 (2.5%)** |
| Beverages | **3379** | **1350 (40.0%)** | **1842 (54.5%)** | **1307 (38.7%)** | **526 (15.6%)** | **9 (0.3%)** |
| Alcohol* | 106 | 106 (100.0%) | 0 | 0 | 0 | 0 |
| Classic  coffee/teas | 314 | 309 (98.4%) | 5 (1.6%) | 3 (1.0%) | 2 (0.6%) | 0 |
| Flavoured  coffee/teas | 984 | 470 (47.8%) | 514 (52.2%) | 256 (26.0%) | 257 (26.1%) | 1 (0.1%) |
| Flavoured  Milk | 176 | 43 (24.4%) | 133 (75.6%) | 30 (17.0%) | 102 (58.0%) | 1 (0.6%) |
| Juices/  Smoothies | 740 | 249 (33.6%) | 491 (66.4%) | 469 (63.4%) | 22 (3.0%) | 0 |
| Kids | 39 | 26 (66.7%) | 13 (33.3%) | 12 (30.8%) | 1 (2.6%) | 0 |
| Milkshakes/  Floats | 205 | 18 (8.8%) | 187 (91.2%) | 38 (18.5%) | 142 (69.3%) | 7 (3.4%) |
| Plain Milk* | 39 | 39 (100.0%) | 0 | 0 | 0 | 0 |
| Soft drinks | 734 | 235 (32.0%) | 499 (68.0%) | 499 (68.0%) | 0 | 0 |
| Water* | 42 | 42 (100.0%) | 0 | 0 | 0 | 0 |
| Desserts | **1334** | **229 (17.2%)** | **1105 (82.8%)** | **463 (34.7%)** | **541 (40.6%)** | **101 (7.6%)** |
| Cookies | 90 | 11 (12.2%) | 79 (87.8%) | 29 (32.2%) | 45 (50.0%) | 5 (5.6%) |
| Donuts | 75 | 20 (26.7%) | 55 (73.3%) | 28 (37.3%) | 20 (26.7%) | 7 (9.3%) |
| Frozen  desserts | 520 | 78 (15.0%) | 442 (85.0%) | 264 (50.8%) | 161 (31.0%) | 17 (3.3%) |
| Kids | 89 | 39 (43.8%) | 50 (56.2%) | 23 (25.8%) | 27 (30.3%) | 0 |
| Misc. | 8 | 0 | 8 (100.0%) | 0 | 7 (87.5%) | 1 (12.5%) |
| Muffins | 63 | 2 (3.2%) | 61 (96.8%) | 17 (27.0%) | 30 (47.6%) | 14 (22.2%) |
| Other  baked goods | 489 | 79 (16.2%) | 410 (83.8%) | 102 (20.9%) | 251 (51.3%) | 57 (11.7%) |
| Entrées | **6848** | **691 (10.1%)** | **6157 (89.9%)** | **2028 (29.6%)** | **3922 (57.3%)** | **207 (3.0%)** |
| Breakfast  Baked goods | 133 | 3 (2.3%) | 130 (97.7%) | 40 (30.1%) | 59 (44.4%) | 31 (23.3%) |
| Breakfast  Egg plates | 237 | 10 (4.2%) | 227 (95.8%) | 10 (4.2%) | 151 (63.7%) | 66 (27.8%) |
| Breakfast  Misc. | 72 | 6 (8.3%) | 66 (91.7%) | 15 (20.8%) | 39 (54.2%) | 12 (16.7%) |
| Breakfast  Sandwich/  Wrap/Toast | 187 | 3 (1.6%) | 184 (98.4%) | 30 (16.0%) | 146 (78.1%) | 8 (4.3%) |
| Burgers | 347 | 3 (0.9%) | 344 (99.1%) | 61 (17.6%) | 281 (81.0%) | 2 (0.6%) |
| Hotdogs | 29 | 0 | 29 (100.0%) | 0 | 29 (100.0%) | 0 |
| Kids | 264 | 48 (18.2%) | 216 (81.8%) | 112 (42.4%) | 96 (36.4%) | 8 (3.0%) |
| Meat only | 123 | 4 (3.3%) | 119 (96.7%) | 32 (26.0%) | 71 (57.7%) | 16 (13.0%) |
| Meat plate | 163 | 13 (8.0%) | 150 (92.0%) | 62 (38.0%) | 82 (50.3%) | 6 (3.7%) |
| Misc. | 15 | 0 | 15 (100.0%) | 0 | 15 (100.0%) | 0 |
| Other  Baked goods | 81 | 1 (1.2%) | 80 (98.8%) | 9 (11.1%) | 70 (86.4%) | 1 (1.2%) |
| Pasta | 219 | 11 (5.0%) | 208 (95.0%) | 86 (39.3%) | 120 (54.8%) | 2 (0.9%) |
| Pizza | 2022 | 236 (11.7%) | 1786 (88.3%) | 411 (20.3%) | 1374 (68.0%) | 1 (0.0%) |
| Poultry only | 260 | 30 (11.5%) | 230 (88.5%) | 102 (39.2%) | 126 (48.5%) | 2 (0.8%) |
| Poultry plate | 195 | 19 (9.7%) | 176 (90.3%) | 87 (44.6%) | 84 (43.1%) | 5 (2.6%) |
| Poutine | 286 | 2 (0.7%) | 284 (99.3%) | 9 (3.1%) | 248 (86.7%) | 27 (9.4%) |
| Salad | 286 | 58 (20.3%) | 228 (79.7%) | 117 (40.9%) | 111 (38.8%) | 0 |
| Sandwiches/  Wraps | 868 | 28 (3.2%) | 840 (96.8%) | 281 (32.4%) | 545 (62.8%) | 14 (1.6%) |
| Seafood  only | 47 | 5 (10.6%) | 42 (89.4%) | 19 (40.4%) | 22 (46.8%) | 1 (2.1%) |
| Seafood  plate | 399 | 132 (33.1%) | 267 (66.9%) | 214 (53.6%) | 53 (13.3%) | 0 |
| Soup | 183 | 31 (16.9%) | 152 (83.1%) | 129 (70.5%) | 23 (12.6%) | 0 |
| Tacos/Burritos | 274 | 19 (6.9%) | 255 (93.1%) | 106 (38.7%) | 149 (54.4%) | 0 |
| Vegetarian | 158 | 29 (18.4%) | 129 (81.6%) | 96 (60.8%) | 28 (17.7%) | 5 (3.2%) |
| Sides | **1438** | **499 (34.7%)** | **863 (60.0%)** | **490 (34.1%)** | **365 (25.4%)** | **8 (0.6%)** |
| Bagels | 62 | 2 (3.2%) | 60 (96.8%) | 27 (43.5%) | 32 (51.6%) | 1 (1.6%) |
| Breads | 181 | 119 (65.7%) | 62 (34.3%) | 46 (25.4%) | 16 (8.8%) | 0 |
| Breakfast | 96 | 36 (37.5%) | 60 (62.5%) | 40 (41.7%) | 20 (20.8%) | 0 |
| Cheese* | 61 | 0 | 0 | 0 | 0 | 0 |
| Fries&  Onion rings | 252 | 46 (18.3%) | 206 (81.7%) | 77 (30.6%) | 124 (49.2%) | 5 (2.0%) |
| Fruit* | 19 | 16 (84.2%) | 3 (15.8%) | 2 (10.5%) | 1 (5.3%) | 0 |
| Kids | 58 | 33 (56.9%) | 25 (43.1%) | 19 (32.8%) | 6 (10.3%) | 0 |
| Meat | 44 | 12 (27.3%) | 32 (72.7%) | 16 (36.4%) | 16 (36.4%) | 0 |
| Misc. | 26 | 15 (57.7%) | 11 (42.3%) | 8 (30.8%) | 3 (11.5%) | 0 |
| Other  baked goods | 41 | 13 (31.7%) | 28 (68.3%) | 18 (43.9%) | 10 (24.4%) | 0 |
| Pasta | 13 | 2 (15.4%) | 11 (84.6%) | 2 (15.4%) | 9 (69.2%) | 0 |
| Potatoes  (non-fried) | 70 | 13 (18.6%) | 57 (81.4%) | 44 (62.9%) | 13 (18.6%) | 0 |
| Poultry | 116 | 15 (12.9%) | 101 (87.1%) | 41 (35.3%) | 58 (50.0%) | 2 (1.7%) |
| Rice | 31 | 14 (45.2%) | 17 (54.8%) | 13 (41.9%) | 4 (12.9%) | 0 |
| Salad | 166 | 93 (56.0%) | 73 (44.0%) | 45 (27.1%) | 28 (16.9%) | 0 |
| Seafood | 42 | 30 (71.4%) | 12 (28.6%) | 7 (16.7%) | 5 (11.9%) | 0 |
| Soup | 68 | 16 (23.5%) | 52 (76.5%) | 42 (61.8%) | 10 (14.7%) | 0 |
| Vegetables | 92 | 39 (42.4%) | 53 (57.6%) | 43 (46.7%) | 10 (10.9%) | 0 |
| Starters | **284** | **34 (12.0%)** | **250 (88.0%)** | **112 (39.4%)** | **137 (48.2%)** | **1 (0.4%)** |
| Breads | 31 | 3 (9.7%) | 28 (90.3%) | 8 (25.8%) | 20 (64.5%) | 0 |
| Cheese* | 11 | 0 | 11 (100.0%) | 1 (9.1%) | 10 (90.9%) | 0 |
| Dips | 26 | 1 (3.8%) | 25 (96.2%) | 4 (15.4%) | 21 (80.8%) | 0 |
| Fries &  Onion rings | 27 | 1 (3.7%) | 26 (96.3%) | 12 (44.4%) | 14 (51.9%) | 0 |
| Meat | 16 | 1 (6.3%) | 15 (93.8%) | 4 (25.0%) | 11 (68.8%) | 0 |
| Misc. | 15 | 1 (6.7%) | 14 (93.3%) | 3 (20.0%) | 10 (66.7%) | 1 (6.7%) |
| Poultry | 41 | 2 (4.9%) | 39 (95.1%) | 11 (26.8%) | 28 (68.3%) | 0 |
| Salad | 12 | 6 (50.0%) | 6 (50.0%) | 3 (25.0%) | 3 (25.0%) | 0 |
| Seafood | 40 | 4 (10.0%) | 36 (90.0%) | 24 (60.0%) | 12 (30.0%) | 0 |
| Soup | 51 | 13 (25.5%) | 38 (74.5%) | 34 (66.7%) | 4 (7.8%) | 0 |
| Vegetables | 14 | 2 (14.3%) | 12 (85.7%) | 8 (57.1%) | 4 (28.6%) | 0 |

Abbreviations: FLIP, Food Label Information and Price database; FOP, Front-of-package; ^*^Categories that meet the exemption criteria outlined in the Canada Gazette II; items in these categories were exempted from further analysis.

**Supplementary Table 5.** The number and percentage of menu items in Menu-FLIP 2020 that would display each ‘high-in’ nutrient-of-concern, if the Canadian FOP labelling regulations were applied to restaurant foods.

|  |  | Nutrient-of-concern | | |
| --- | --- | --- | --- | --- |
| Menu Category | Total n | Sodium | Sugars | Saturated Fat |
| Overall | **13283** | **6972 (52.5%)** | **3135 (23.6%)** | **6253 (47.1%)** |
| Beverages | **3379** | 21 (0.6%) | 1779 (52.6%) | 586 (17.3%) |
| Alcohol^*^ | 106 | 0 | 0 | 0 |
| Classic coffee/teas | 314 | 0 | 2 (0.6%) | 5 (1.6%) |
| Flavoured coffee/teas | 984 | 1 (0.1%) | 471 (47.9%) | 301 (30.6%) |
| Flavoured milk | 176 | 1 (0.6%) | 127 (72.2%) | 109 (61.9%) |
| Juices/Smoothies | 740 | 7 (0.9%) | 482 (65.1%) | 24 (3.2%) |
| Kids | 39 | 1 (2.6%) | 11 (28.2%) | 2 (5.1%) |
| Milkshakes/Floats | 205 | 11 (5.4%) | 187 (91.2%) | 145 (70.7%) |
| Plain Milk^*^ | 39 | 0 | 0 | 0 |
| Soft drinks | 734 | 0 | 499 (68.0%) | 0 |
| Water^a^ | 42 | 0 | 0 | 0 |
| Desserts | **1334** | **175 (13.1%)** | **958 (71.8%)** | **715 (53.6%)** |
| Cookies | 90 | 7 (7.8%) | 52 (57.8%) | 75 (83.3%) |
| Donuts | 75 | 11 (14.7%) | 28 (37.3%) | 50 (66.7%) |
| Frozen desserts | 520 | 17 (3.3%) | 433 (83.3%) | 187 (36.0%) |
| Kids | 89 | 2 (2.2%) | 42 (47.2%) | 33 (37.1%) |
| Misc. | 8 | 1 (12.5%) | 8 (100.0%) | 8 (100.0%) |
| Muffins | 63 | 36 (57.1%) | 60 (95.2%) | 23 (36.5%) |
| Other baked goods | 489 | 101 (20.7%) | 335 (68.5%) | 339 (69.3%) |
| Entrées | **6848** | **5760 (84.1%)** | **334 (4.9%)** | **4399 (64.2%)** |
| Breakfast Baked goods | 133 | 79 (59.4%) | 96 (72.2%) | 76 (57.1%) |
| Breakfast Egg plates | 237 | 221 (93.2%) | 68 (28.7%) | 221 (93.2%) |
| Breakfast Misc. | 72 | 53 (73.6%) | 20 (27.8%) | 56 (77.8%) |
| Breakfast  Sandwich/Wrap/Toast | 187 | 178 (95.2%) | 13 (7.0%) | 155 (82.9%) |
| Burgers | 347 | 321 (92.5%) | 3 (0.9%) | 305 (87.9%) |
| Hotdogs | 29 | 29 (100.0%) | 0 | 29 (100.0%) |
| Kids | 264 | 194 (73.5%) | 21 (8.0%) | 113 (42.8%) |
| Meat only | 123 | 95 (77.2%) | 17 (13.8%) | 110 (89.4%) |
| Meat plate | 163 | 139 (85.3%) | 8 (4.9%) | 97 (59.5%) |
| Misc. | 15 | 15 (100.0%) | 0 | 15 (100.0%) |
| Other baked goods | 81 | 74 (91.4%) | 1 (1.2%) | 77 (95.1%) |
| Pasta | 219 | 202 (92.2%) | 7 (3.2%) | 123 (56.2%) |
| Pizza | 2022 | 1624 (80.3%) | 1 (0.0%) | 1537 (76.0%) |
| Poultry only | 260 | 222 (85.4%) | 9 (3.5%) | 129 (49.6%) |
| Poultry plate | 195 | 169 (86.7%) | 9 (4.6%) | 92 (47.2%) |
| Poutine | 286 | 280 (97.9%) | 27 (9.4%) | 279 (97.6%) |
| Salad | 286 | 208 (72.7%) | 8 (2.8%) | 123 (43.0%) |
| Sandwiches/Wraps | 868 | 838 (96.5%) | 15 (1.7%) | 560 (64.5%) |
| Seafood only | 47 | 36 (76.6%) | 1 (2.1%) | 29 (61.7%) |
| Seafood plate | 399 | 262 (65.7%) | 2 (0.5%) | 56 (14.0%) |
| Soup | 183 | 152 (83.1%) | 0 | 23 (12.6%) |
| Tacos/Burritos | 274 | 249 (90.9%) | 0 | 155 (56.6%) |
| Vegetarian | 158 | 120 (75.9%) | 8 (5.1%) | 39 (24.7%) |
| Sides | **1438** | **776 (54.0%)** | **56 (3.9%)** | **412 (28.7%)** |
| Bagels | 62 | 60 (96.8%) | 1 (1.6%) | 33 (53.2%) |
| Breads | 181 | 52 (28.7%) | 0 | 26 (14.4%) |
| Breakfast | 96 | 35 (36.5%) | 17 (17.7%) | 28 (29.2%) |
| Cheese^*^ | 61 | 0 | 0 | 0 |
| Fries & Onion rings | 252 | 199 (79.0%) | 15 (6.0%) | 126 (50.0%) |
| Fruit^*^ | 19 | 0 | 3 (15.8%) | 1 (5.3%) |
| Kids | 58 | 20 (34.5%) | 4 (6.9%) | 7 (12.1%) |
| Meat | 44 | 29 (65.9%) | 0 | 19 (43.2%) |
| Misc. | 26 | 11 (42.3%) | 0 | 3 (11.5%) |
| Other baked goods | 41 | 22 (53.7%) | 1 (2.4%) | 15 (36.6%) |
| Pasta | 13 | 11 (84.6%) | 0 | 9 (69.2%) |
| Potatoes (non-fried) | 70 | 47 (67.1%) | 1 (1.4%) | 22 (31.4%) |
| Poultry | 116 | 97 (83.6%) | 4 (3.4%) | 62 (53.4%) |
| Rice | 31 | 17 (54.8%) | 0 | 4 (12.9%) |
| Salad | 166 | 68 (41.0%) | 4 (2.4%) | 29 (17.5%) |
| Seafood | 42 | 12 (28.6%) | 0 | 5 (11.9%) |
| Soup | 68 | 51 (75.0%) | 0 | 11 (16.2%) |
| Vegetables | 92 | 45 (48.9%) | 6 (6.5%) | 12 (13.0%) |
| Starters | **284** | **240 (84.5%)** | **8 (2.8%)** | **141 (49.6%)** |
| Breads | 31 | 27 (87.1%) | 0 | 21 (67.7%) |
| Cheese^*^ | 11 | 11 (100.0%) | 0 | 10 (90.9%) |
| Dips | 26 | 25 (96.2%) | 0 | 21 (80.8%) |
| Fries & Onion rings | 27 | 24 (88.9%) | 2 (7.4%) | 14 (51.9%) |
| Meat | 16 | 14 (87.5%) | 0 | 12 (75.0%) |
| Misc. | 15 | 14 (93.3%) | 1 (6.7%) | 11 (73.3%) |
| Poultry | 41 | 37 (90.2%) | 3 (7.3%) | 27 (65.9%) |
| Salad | 12 | 4 (33.3%) | 1 (8.3%) | 4 (33.3%) |
| Seafood | 40 | 35 (87.5%) | 0 | 13 (32.5%) |
| Soup | 51 | 37 (72.5%) | 0 | 5 (9.8%) |
| Vegetables | 14 | 12 (85.7%) | 1 (7.1%) | 3 (21.4%) |

Abbreviations: FLIP, Food Label Information and Price database; FOP, Front-of-package; ^*^Categories that meet the exemption criteria outlined in the Canada Gazette II; items in these categories were exempted from further analysis.

**Supplementary Table 6.** Sensitivity analysis of the percentage of menu items with missing serving size in Menu-FLIP 2020 that would display a ‘high-in’ FOP symbol determined using an assessment that was based on major menu categories, where beverages, entrées and starters were evaluated under the 30% DV thresholds based on their mean serving sizes being ≥200g; desserts and sides were evaluated under the 15% DV thresholds as their mean serving sizes were smaller. Difference indicates the discrepancy between the original analysis and the sensitivity analysis.


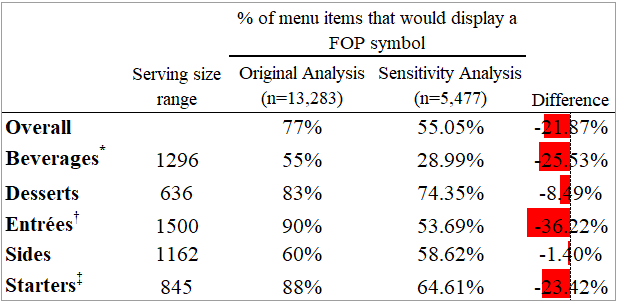


^*^Assessing all beverages under the same threshold (30%) resulted in a significant underestimation as it may have allowed beverages with smaller serving sizes to be assessed against a higher (i.e., less stringent) threshold; ^†^Assessing all entrées under the same threshold (30%) resulted in a significant underestimation as it may have allowed entrées with smaller servings sizes to be assessed against a higher (i.e., less stringent) threshold; ^‡^Assessing all starters under the same threshold (30%) resulted in an underestimation as it may have allowed starters with smaller serving sizes to be assessed against a higher (i.e., less stringent) threshold.
